# Magnolol additive improves growth performance of *Linwu* ducklings by modulating antioxidative status

**DOI:** 10.1371/journal.pone.0259896

**Published:** 2021-12-31

**Authors:** Qian Lin, Yang Liu, Simin Peng, Chunjie Liu, Tuo Lv, Liping Liao, Yinghui Li, Yanzhou Wang, Zhiyong Fan, Weiguo Wu, Jianguo Zeng, Huajiao Qiu, Xi He, Qiuzhong Dai

**Affiliations:** 1 Institute of Bast Fiber Crops, Chinese Academy of Agricultural Sciences, Changsha, Hunan, China; 2 College of Animal Science and Technology, Hunan Agricultural University, Changsha, Hunan, China; 3 College of Food Science and Technology, Hunan Agricultural University, Changsha, Hunan, China; 4 College of Horticulture, Hunan Agricultural University, Changsha, Hunan, China; Sejong University, REPUBLIC OF KOREA

## Abstract

Magnolol is a bioactive polyphenolic compound commonly found in *Magnolia officinalis*. The aim of this study is to clarify the contribution of the magnolol additive on the growth performance of *Linwu* ducklings aging from 7 to 28 d, comparing to the effects of antibiotic additive (colistin sulphate). A total of 325, 7-d-old ducklings were assigned to 5 groups. Each group had 5 cages with 13 ducklings in each cage. The ducklings in different groups were fed with diets supplemented with 0, 100, 200 and 300 mg/kg magnolol additive (MA) (Control, MA100, MA200 and MA300) and 30 mg/kg colistin sulphate (CS30) for 3 weeks, respectively. Parameters regarding to the growth performance, intestinal mucosal morphology, serum biochemical indices, antioxidant and peroxide biomarkers and the expression levels of antioxidant-related genes were evaluated by one way ANOVA analysis. The results showed that 30 mg/kg colistin sulphate, 200 and 300 mg/kg magnolol additive improved the average final weight (*P* = 0.045), average daily body weight gain (*P* = 0.038) and feed/gain ratios (*P* = 0.001) compared to the control group. 200 and 300 mg/kg magnolol additive significantly increased the villus height/crypt depth ratio of ileum, compared to the control and CS30 groups (*P* = 0.001). Increased serum level of glucose (*P* = 0.011) and total protein (*P* = 0.006) were found in MA200 or MA300 group. In addition, comparing to the control and CS30 groups, MA200 or MA300 significantly increased the levels of superoxide dismutase (*P* = 0.038), glutathione peroxidase (*P* = 0.048) and reduced glutathione (*P* = 0.039) in serum. Moreover, the serum and hepatic levels of 8-hydroxy-2’-deoxyguanosine (*P* = 0.043 and 0.007, respectively) were lower in all MA groups compared to those of the control and CS30 group. The hepatic mRNA expression levels of *superoxide dismutase-1*, *catalase* and *nuclear factor erythroid-2-related factor 2/erythroid-derived CNC-homology factor* were also increased significantly in MA200 and MA300 groups (*P* < 0.05). Taken together, these data demonstrated that MA was an effective feed additive enhancing the growth performance of *Linwu* ducklings at 7 to 28 d by improving the antioxidant and intestinal mucosal status. It suggested that MA could be a potential ingredient to replace the colistin sulphate in diets.

## Introduction

Oxidative stress is considered to be the imbalance between the oxidation and antioxidant defense system within organisms, which results in lipid peroxidation and oxidative damages to DNA and proteins [1]. Due to the sudden transition process from chorioallantoic to pulmonary respiration, the metabolic rate and oxygen consumption increase rapidly after hatching to meet the demands of endothermy and locomotion, causing young birds facing severe oxidative stress during their birth and early growth stages [2, 3]. On the other hand, the abuse of antibiotics as growth promoting feed additives has increased the risk of drug resistance [4]. In recent years, it has been growing rapidly that the consumer demand safe, healthy and high-quality poultry products. These situations required breeders to develop healthy and sustained poultry industry by reducing the antibiotics use. Phytochemicals were potential replacers to the antibiotics. Many phytochemicals had profound impacts on the growth performance and antioxidant ability of animals [5], such as resveratrol [6], oregano essential oil [7] and aloe vera [8].

Magnolol is a 4-allyl-2-(5-allyl-2-hydroxy-phenyl) phenol rich in the bark of the Houpu magnolia (*Magnolia officinalis)*. Accumulated data revealed that magnolol had anti-inflammatory [9], antineoplastic [10], anti-stress [11] and antidiarrheic effect [12]. Especially, the remarkable antioxidant effect of magnolol was demonstrated by *in vitro* and *in vivo* assays [13]. Magnolol could scavenge the content of DPPH (1,1-Diphenyl-2-picrylhydrazyl), ABTS (2,2′-Azinobis [3-ethylbenzothiazoline-6-sulfonic acid)], ferric ions (Fe^3+^), superoxide anion and hydroxyl radicals *in vitro* [14–17]. It was estimated that the antioxidant activity of magnolol was higher than that of α-tocopherol [18]. Additionally, it was reported that magnolol could protect the organs and tissues from injuries by scavenging free radicals and activating the signaling pathways that mediate the expressions of antioxidant or detoxifying enzymes in rat [19] and mouse [20].

Even though magnolol was studied in animal models including mice [21] and laying hens [22], the effects of magnolol on ducks were rarely reported. *Linwu* duck, a major indigenous breed of ducks in China, were used in the present study. *Linwu* duck is an indigenous duck breed widely farmed in South China. It is famous in local and global markets for the unique meat flavor and texture comparing to other main commercial duck breeds, such as Peking ducks. The present research aimed to clarify the hypothesis that magnolol could be beneficial to the *Linwu* ducklings as feed additive, and evaluate the potential of magnolol as an antibiotic replacer by comparing the effects of magnolol with that of antibiotic agent.

## Materials and methods

### Ethical statement

All the experimental procedures were conducted in accordance with the Chinese guidelines for animal welfare and approved by the Animal Care and Use Committee, Institute of Bast Fiber Crops, Chinese Academy of Agricultural Sciences (Hunan Province, Changsha, China). The authorization number was 2020007.

### Birds, diets, and experimental design

Three hundred and twenty-five female *Linwu* ducklings at 1 d of age were obtained from Hunan Shunhua Duck Industrial Development Company. Magnolol additive (**MA**) was extracted from *Magnolia officinalis* at the National Research Center of Engineering Technology for Utilization of Functional Ingredients from Botanicals. Briefly, magnolol was extracted with ethanol at 70% volume fraction, with the solid/liquid proportion of 0.26 g/mL. The extracting temperature was 90°C for and the extracting time period lasted 24 hours. The extraction was separated by silica gel column chromatography. The silica gel column size was 2.5 by 50 cm with 100 to 200 meshes, and was pretreated with ethanol to eliminate the air within, then heated in 120°C for 24 hours. The purity of extracted magnolol was identified as 98.1% by HPLC at wavelength of 292 nm (Agilent 1100). The flowing phase for the HPLC was 80% ethanol and 20% water with the flowing speed at 0.8 mL/min. The size of the filter membrane was 0.22 μm. The ducklings were supplied with *ad libitum* accessing to feed and water throughout the 21 d trial period. The average temperature during the trial period was 27.1°C. After a 1-wk adaptation period, the ducklings were individually weighed and divided into 5 groups making sure that the average initial weight among groups were not significant difference. Each group (65 *Linwu* ducklings) was further subdivided into 5 cages with 13 ducklings in each cage, and the dimension of each cage was 120 cm × 120 cm. Group one received a basal diet (**BD**) as the control group. Group two, three and four received BD supplemented with 100, 200 and 300 mg/kg diet respectively, which were named as MA100, MA200, MA300, respectively. Group five received BD supplemented with 30 mg/kg colistin sulphate (**CS**) (Guangzhou Xingda Animal’s Pharmaceutical Company, Guangdong, China), which was named CS30. The BD was formulated in according to Nutrient Requirements of Meat-type Duck (China, NY/T 2122–2012) and the Nutrient Requirements of Ducks [23] (Table 1).

**Table 1:** Composition and nutrient levels of basal diet (air-dry basis, %).

Item	Ingredients	Item	Nutrient levels^2^
Corn	60.00	Metabolizable energy, kcal/kg	2800.00
Soybean meal	22.19	Dry matter	86.99
Wheat bran	3.50	Crude protein	19.00
Wheat middling	3.05	Crude lipid	3.15
Cottonseed meal	3.00	Crude fiber	2.88
Rapeseed meal	3.00	Lysine	1.10
Calcium hydrophosphate	1.52	Calcium	0.90
Limestone	1.33	Methionine+Cysteine	0.77
1% Premix^1^	1.00	Threonine	0.71
Soybean oil	0.66	Total phosphorus	0.65
Sodium chloride	0.30	Methionine	0.45
78% *L*- Lys	0.27	Available phosphorus	0.38
98.5% *DL*- Met	0.18	Salt	0.34
Total	100.00	Tryptophane	0.22

^1^The premix provides following per kg diets: VA 12 000 IU, VD_3_ 2 500 IU, VE 20 mg, VK_3_ 3 mg, VB_1_ 3 mg, VB_2_ 8 mg, VB_6_ 7 mg, VB_12_ 0.03 mg, *D*-pantothenic acid 20 mg, nicotinic acid 50 mg, biotin 0.1 mg, folic acid 1.5 mg, Cu (as copper sulfate) 9 mg, Zn (as zinc sulfate) 110 mg, Fe (as ferrous sulfate) 100 mg, Mn (as manganese sulfate) 100 mg, Se (as sodium selenite) 0.16 mg, I (as potassium iodide) 0.6 mg.

^2^Nutrient levels are calculated values.

### Sample collection

Body weight of *Linwu* duckling was individually measured at the end of the trial (28 d). Feed intake per cage was recorded daily. The average daily feed intake (**ADFI**), average daily body weight gain (**ADG**) and feed/gain ratios (**F/G**) were calculated according to the data from each cage. On 28 d, 10 ducklings from each group (2 ducklings in each cage) were slaughtered by cervical dislocation after 12-h fasting. The serum was separated by centrifugation at 3000 × g for 15 min at 4°C and stored at -20°C until analysis. The liver was immediately removed from the carcass, frozen in liquid nitrogen, and stored at -80°C until analysis. The small intestine was promptly moved out and divided into 3 parts: duodenum, jejunum and ileum. A 2 cm-segment of intestine was cut from the midpoint of the duodenum, jejunum, ileum. These intestinal tissue samples were lightly flushed with physiological saline (154 mmol/L), blotted dry with filter paper and fixed into 10% neutral buffered formalin for further analysis of intestinal mucosal morphology.

### Measurement of intestinal mucosal morphology

Measurement of intestinal mucosal morphology was described previously according to Jiang et al. [24]. Briefly, the 2 cm-intestinal tissue samples of the duodenum, jejunum, ileum were embedded in paraffin. A microtome (RM-2235, Leica microsystems AG., Hessen, Germany) was used to make 5 or 6 μm slices that were mounted in glass slides and subsequently stained with hematoxylin and eosin. Finished slides were observed under an Olympus Van-Ox S microscope (Opelco, Washington, DC) and 10 typical microscopic fields were selected for photo taking. Villus height and crypt depth from each slide were determined, using an image analysis system (Image-Pro, Media Cybernetics, Inc., Silver Springs, MD). The height of 10 intact villi and the depth of their associated crypts were measured in each slide.

### Measurement of serum biochemical indices

All the serum and hepatic parameters were tested three times and the means were calculated for the following statistical analysis. The serum levels of glucose (**GLU**), triglyceride (**TG**), total cholesterol (**TCHO**), uric acid (**UA**), urea nitrogen (**BUN**), creatinine (**CR**), total protein (**TP**), albumin (**ALB**), globin (**GLB**) and the activities of alanine amino transferase (**ALT**) and aspartate amino transferase (**AST**) were measured with the commercial assay kits (Guilin Urit Medical Electronics Co., LTD, Guilin, China) by an automatic biochemical analyzer (URIT-8000, Guilin Urit Medical Electronics Co., LTD, Guilin, China), as described previously [25].

### Measurement of serum antioxidant biomarkers

The serum levels of reduced glutathione (**GSH**) and the activities of superoxide dismutase (**SOD**), catalase (**CAT**), glutathione peroxidase (**GPX**) and total antioxidant capacity (**T-AOC**) were determined by the commercial kits (Nanjing Jiancheng Bioengineering Institute, Nanjing, China) with an automated fluorescence instrument (Thermo Fisher Scientific, Waltham, MA). Briefly, the GSH concentration was measured according to the methods described previously with minor modification [26]. The activity of SOD was determined at 450 nm optical density by the nitrite formation method. The CAT activity was determined by incubating samples with 0.05 mol/L H_2_O_2_, and then measuring at 405 nm optical density by the ammonium molybdate method. The GPX activity was assayed by quantifying the oxidation rate of GSH to oxidized glutathione at 412 nm optical density. The T-AOC was determined by the level of ferric reducing ability of plasma at 520 nm optical density.

### Measurement of serum and hepatic peroxide biomarkers

The homogenate of hepatic sample was prepared as described previously [27]. Briefly, 0.3 g of hepatic samples were homogenized with ice-cold physiological saline (1:9, wt/vol), using an Ultra-Turrax homogeniser (Tekmar Company, Cincinatti, OH) centrifuged at 4000 × g at 4°C for 15 min. The supernatant was used for the following assays. The serum and hepatic levels of malonaldehyde (**MDA**) were determined using commercial kits (Nanjing Jiancheng Bioengineering Institute, Nanjing, China), by thiobarbituric acid reaction method at 532 nm optical density. The serum and hepatic protein carbonyl (**PC**) levels was determined, using an ELISA kit (OxiSelect Protein Carbonyl ELISA Kit; Cell Biolabs, San Diego, CA, USA) and the concentrations of PC were expressed as nmol/mg protein. The serum and hepatic levels of 8-hydroxy-2’-deoxyguanosine (**8-OHdG**) were quantified by a specific ELISA kit (Abcam, Cambridge, U.K.). All results were normalized to total protein concentration of each sample for comparison.

### Quantification of mRNA expression by real-time PCR

Total RNA from the liver was isolated using Trizol reagent (TaKaRa, Tokyo, Japan), then treated with DNase I (Thermo Fisher Scientific Inc., USA). cDNA was synthesized from 1 μg of RNA with a RevertAid First Strand cDNA Synthesis Kit (Thermo Fisher Scientific Inc., USA) according to the manufacturer’s instructions. Based on the cloned complete sequences (https://www.ncbi.nlm.nih.gov/genbank/) of nuclear factor erythroid-2-related factor 2/erythroid-derived CNC homology factor (***Nrf2/ECH***), kelch-like ECH-associated protein 1 (***Keap-1***), heme oxygenase-1 (***HO-1***), catalase (***CAT***), glutathione S-transferase α3 (***GSTα3***), superoxide dismutase-1 (***SOD1***), manganese superoxide dismutase-2 (***MnSOD***), glutathione peroxidase-1 (***GPX1***), glutathione peroxidase-4 (***GPX4***) and *β*-actin from *Anas platyrhynchos*, primer pairs were designed with Primer 5.0 for quantitative real-time PCR (Table 2). The *β*-actin gene was used as the housekeeping gene, whose stability was confirmed via the comparative ΔCt method following the discription by Chen et al. [28]. All primers were synthesized and purified by Sangon Biotech Co. Ltd (Shanghai, China). Reaction volume of 20 μL mixture contained 10 μL Power SYBR Green PCR Master Mix (Applied Biosystems, Foster City, CA, USA), 1 μL cDNA template, 1 μL of each of the upstream and downstream primers, and 7 μL sterilized deionized water. All sample analyses were carried out in triplicate and the average values were indexed. The target gene expression was normalized to that of the selected reference gene, and the relative gene expression was calculated using 2^−ΔΔCt^ method [29]. The mRNA levels were expressed as the fold change relative to the mean value of the control group, which was arbitrarily defined as 1.0.

**Table 2:** Oligonucleotides sequence used for real-time quantitative PCR.

Primer name	Sequences of the primer pair	GenBank accession NO.	Fragment length, bp
*β-Actin* sense	5’-AGTACCCCATTGAACACGGT-3’	EF667345	197
*β-Actin* antisense	5’-ATACATGGCTGGGGTGTTGA-3’		
*GPX1* sense	5’-TTCGAGAAGTGCGAGGTGAA-3’	KU048803	156
*GPX1* antisense	5’-GTTCCAGGAGATGTCGTTGC-3’		
*GPX4* sense	5’-TTTGCTGAGAACTACGGGGT-3’	KU048804	192
*GPX4* antisense	5’-GGGGCTGTATCTCTTCACCA-3’		
*GST α3* sense	5’-AGAGAGCCCTGATCGACATG-3’	KU048805	177
*GST α3* antisense	5’-AGTCTTGGCCGTGTTGTTTC-3’		
*HO-1* sense	5’-TGCCTACACTCGCTATCTGG-3’	KU048806	183
*HO-1* antisense	5’-AGGTCCATCTCAAGGGCATT-3’		
*CAT* sense	5’-AATGTGCGTGACTGACAACC-3’	KU048802	196
*CAT* antisense	5’-ACGTTCATCCTCCTTCAGCA-3’		
*Keap-1* sense	5’-CAGTCCTTGGGCTACTTGGA-3’	KU048807	197
*Keap-1* antisense	5’-CGGTTGGTCATGGGGTTGTA-3’		
*MnSOD* sense	5’-GACCTGCCCTACGACTATGG-3’	KU048809	167
*MnSOD* antisense	5’-TGAAGTGACACCTGAGCTGT-3’		
*SOD1* sense	5’-TGGACCAAAGGATGCAGAGA-3’	KU048808	200
*SOD1* antisense	5’-CATTCCCAGTTAGCGTGCTC-3’		
*Nrf2/ECH* sense	5’-CGCCTTGAAGCTCATCTCAC-3’	KM109969	176
*Nrf2/ECH* antisense	5’-TTCTTGCCTCTCCTGCGTAT-3’		

*GPX1* = glutathione peroxidase-1; *GPX4* = glutathione peroxidase-4; *GSTα3* = glutathione S-transferase α3; *HO-1* = heme oxygenase-1; *CAT* = catalase; *Keap-1* = kelch-like ECH-associated protein 1; *MnSOD* = manganese superoxide dismutase; *SOD1* = superoxide dismutase-1; *Nrf2/ECH* = nuclear factor erythroid-2-related factor 2/erythroid-derived CNC homology factor.

### Statistical analysis

Data were analyzed using Statistical Package for the Social Sciences 19.0 (IBM, Armonk, New York). One-way ANOVA model was performed to test all data. Replicate was used as the experimental unit. Results were presented as means and pooled standard errors of the means (**SEM**). When the main effect was significant, the differences among means were further determined using Duncan’s multiple range. Differences between means of all groups were considered significant at *P* < 0.05.

## Results

### Growth performance

Effects of dietary MA and CS on growth performance of *Linwu* ducklings were showed in Table 3. Though the ADFI among 5 groups are similar (*P* = 0.741), dietary supplementation of CS and MA at intermediate or high level (200 or 300 mg/kg) caused significant improvement in terms of average final weight (*P* = 0.045), ADG (*P* = 0.038), and F/G (*P* = 0.001) compared to the control group. Low level of MA (100 mg/kg) also showed significant lower F/G than the control group (*P* ≤ 0.05).

**Table 3:** Effect of dietary magnolol at different levels on growth performance of *Linwu* ducks.

Items^1^	C	MA100	MA200	MA300	CS30	SEM	*P*-value
Average initial weight, g	91.81	92.26	92.20	92.11	92.19	0.096	0.642
Average final weight, g	481.84^b^	485.03^ab^	488.54^a^	487.46^a^	486.65^a^	0.780	0.045
ADG, g	18.57^b^	18.70^ab^	18.87^a^	18.82^a^	18.78^a^	0.035	0.038
ADFI, g	55	54	54	54	54	0.120	0.741
F/G	2.93^a^	2.89^b^	2.87^b^	2.88^b^	2.88^b^	0.006	0.001

ADG = average daily body weight gain; ADFI = average daily feed intake; F/G = feed/gain ratios.

^a-b^In the same row, values with different small letter superscripts mean significant difference (*P* < 0.05).

^1^C: control, basal diet; MA100: basal diet + 100 mg magnolol additive/kg diet; MA200: basal diet + 200 mg magnolol additive/kg diet; MA300: basal diet + 300 mg magnolol additive/kg diet; CS30: basal diet + 30 mg antibiotic additive/kg diet.

### Intestinal mucosal morphology

**Fig 1:**
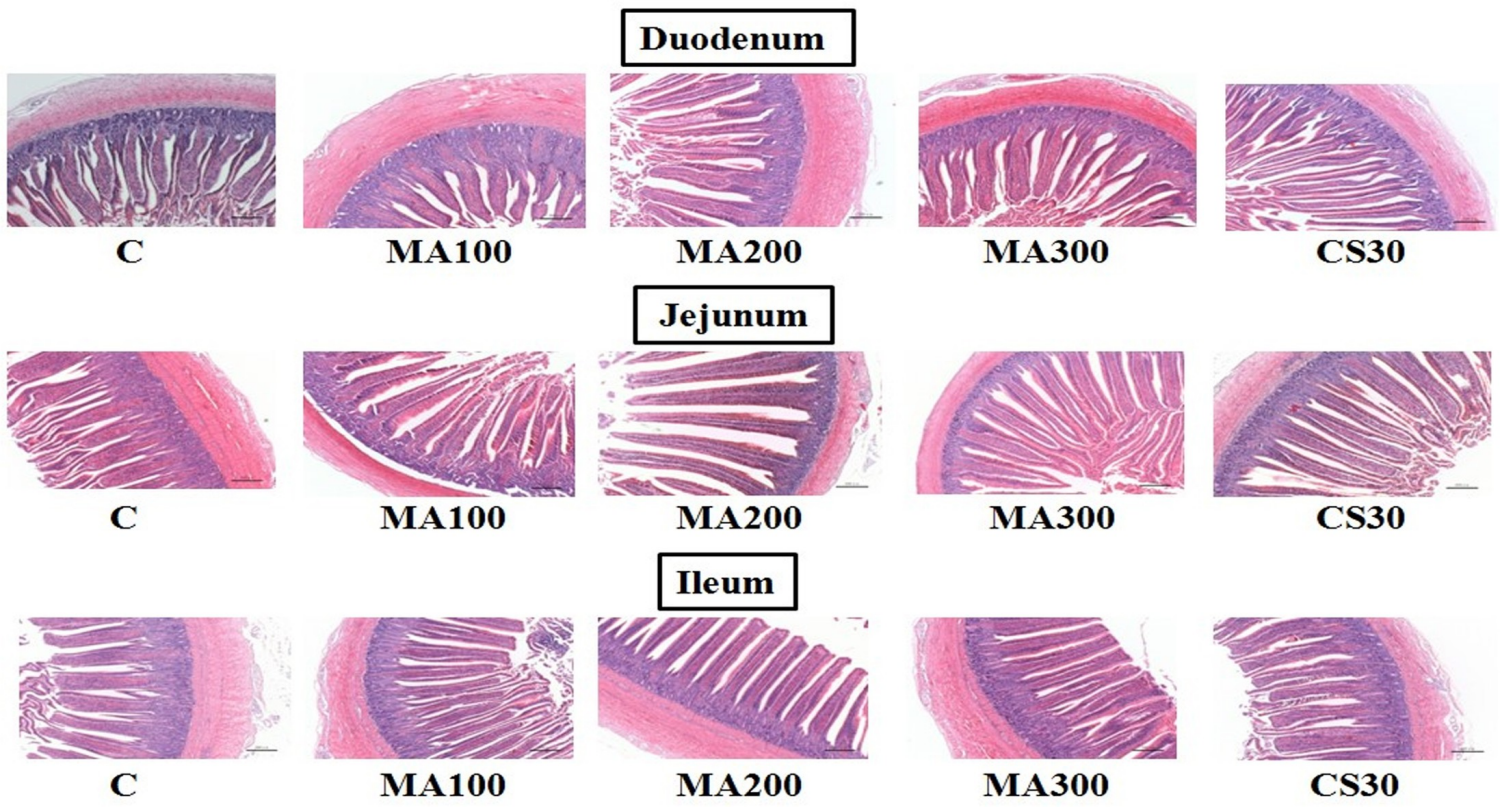
Effect of dietary magnolol at different levels on the intestinal mucosal morphology of 28 d *Linwu* ducklings (40 ×). C: control, basal diet; MA100: basal diet + 100 mg magnolol additive/kg diet; MA200: basal diet + 200 mg magnolol additive/kg diet; MA300: basal diet + 300 mg magnolol additive/kg diet; CS30: basal diet + 30 mg antibiotic additive/kg diet.

The mucosal morphologies of intestine segments including duodenum, jejunum and ileum were observed afterHE staining. Ducklings fed with 200 mg/kg MA had more complete intestinal mucosal than that with control and CS30 groups in duodenum, jejunum and ileum (Fig 1). MA200 and MA300 significantly increased the villus height/crypt depth ratio of ileum compared to the control and CS30 groups (*P* = 0.001) (Table 4). In addition, there were no significant different on villus height and crypt depth in duodenum, jejunum or ileum among groups (*P* > 0.05) (Table 4).

**Table 4:** Effects of dietary magnolol at different levels on the intestinal mucosal morphology of 28 d *Linwu* ducklings.

Items^1^	C	MA100	MA200	MA300	CS30	SEM	*P*-value
Duodenum							
Villus height, μm	694.99	696.20	708.66	704.31	711.16	8.327	0.971
Crypt depth, μm	178.88	175.90	174.11	170.92	172.30	2.125	0.812
Villus height/Crypt depth	3.89	3.97	4.07	4.15	4.14	0.066	0.715
Jejunum							
Villus height, μm	626.10	634.83	646.87	638.19	649.77	12.176	0.980
Crypt depth, μm	167.81	162.15	158.90	160.06	162.57	1.663	0.524
Villus height/Crypt depth	3.74	3.92	4.07	3.99	4.02	0.083	0.795
Ileum							
Villus height, μm	650.69	662.84	667.38	657.71	652.34	5.306	0.879
Crypt depth, μm	157.65	150.31	152.75	154.75	158.06	1.533	0.507
Villus height/Crypt depth	4.13^c^	4.25^bc^	4.41^a^	4.37^ab^	4.13^c^	0.030	0.001

^a-c^In the same row, values with different small letter superscripts mean significant difference (*P* < 0.05).

^1^C: control, basal diet; MA100: basal diet + 100 mg magnolol additive/kg diet; MA200: basal diet + 200 mg magnolol additive/kg diet; MA300: basal diet + 300 mg magnolol additive/kg diet; CS30: basal diet + 30 mg antibiotic additive/kg diet.

### Serum biochemical indices

Dietary MA exhibited non-significant effects on serum biochemical parameters except the glucose and total protein levels in this study (Table 5). MA200 significantly increased the serum levels of glucose (*P* = 0.011) and total protein (*P* = 0.006) compared with the control and CS30 groups. MA 300 significantly increased the serum levels of glucose compared with the control and CS 30 groups (*P* = 0.011), and increased total protein level compared with the control group (*P* = 0.006).

**Table 5:** Effect of different magnolol levels in diet on serum biochemical indices of 28 d *Linwu* ducks.

Items^1^	C	MA100	MA200	MA300	CS30	SEM	*P*-value
GLU, mmol/L	9.83^c^	10.05^bc^	10.88^ab^	11.08^a^	9.89^c^	0.158	0.011
TG, mmol/L	0.63	0.68	0.69	0.71	0.64	0.013	0.223
TCHO, mmol/L	5.58	6.08	6.11	6.33	5.93	0.092	0.104
UA, μmol/L	246.60	256.60	266.20	261.20	258.00	5.092	0.834
BUN, mmol/L	0.52	0.60	0.62	0.57	0.60	0.013	0.069
CR/(μmol/L)	35.80	40.20	38.40	38.20	35.40	0.619	0.065
TP, g/L	21.53^c^	23.29^abc^	24.69^a^	23.68^ab^	21.91^bc^	0.337	0.006
ALB, g/L	9.33	10.08	10.97	10.41	9.56	0.231	0.164
GLB, g/L	12.20	13.22	13.72	13.27	12.35	0.216	0.109
ALB/GLB	0.77	0.76	0.80	0.79	0.78	0.021	0.987
ALT, U/L	64.20	59.00	61.40	61.00	62.00	1.112	0.720
AST, U/L	79.40	76.00	78.60	78.40	82.20	1.292	0.701
AST/ALT	1.24	1.29	1.29	1.29	1.34	0.026	0.858

GLU = glucose; TG = triglyceride; TCHO = total cholesterol; UA = uric acid; BUN = urea nitrogen; CR = creatinine; TP = total protein; ALB = albumin; GLB = globin; ALT = alanine amino transferase; AST = aspartate amino transferase.

^a-c^In the same row, values with different small letter superscripts mean significant difference (*P* < 0.05).

^1^C: control, basal diet; MA100: basal diet + 100 mg magnolol additive/kg diet; MA200: basal diet + 200 mg magnolol additive/kg diet; MA300: basal diet + 300 mg magnolol additive/kg diet; CS30: basal diet + 30 mg antibiotic additive/kg diet.

### Serum antioxidant biomarkers

MA100 showed significantly higher GPX (*P* = 0.048) and GSH (*P* = 0.039) levels in serum compared with the CS30 group. MA 200 showed significantly higher GPX levels in serum compared with the control group (*P* = 0.048), and significantly higher SOD (*P* = 0.038), GPX (*P* = 0.048), and GSH (*P* = 0.039) levels in serum compared with the CS30 group. MA30 showed significantly higher SOD (*P* = 0.038) level in serum compared with both the control and CS30 groups, and significantly higher GSH (*P* = 0.039) level in serum compared with the CS30 group (Table 6). However, there were no significant differences (*P* > 0.05) among all groups on serum levels of CAT and T-AOC.

**Table 6:** Effect of dietary magnolol at different levels on serum antioxidant indices of 28 d *Linwu* ducks.

Items^1^	C	MA100	MA200	MA300	CS30	SEM	*P*-value
SOD, U/ml	86.73^bc^	88.15^abc^	95.82^ab^	97.51^a^	85.11^c^	1.679	0.038
GPX, U/ml	600.00^bc^	647.1^ab^	656.07^a^	638.93^abc^	588.57^c^	9.140	0.048
CAT, U/ml	1.39	1.59	1.66	1.52	1.54	0.063	0.754
GSH, ng/ml	429.72^ab^	440.12^a^	461.66^a^	450.76^a^	391.35^b^	8.098	0.039
T-AOC, U/ml	10.77	12.57	13.29	13.28	11.83	0.345	0.083

SOD = superoxide dismutase; GPX = glutathione peroxidase; CAT = catalase; GSH = reduced glutathione; T-AOC = total antioxidant capacity.

^a-c^In the same row, values with different small letter superscripts mean significant difference (*P* < 0.05).

^1^C: control, basal diet; MA100: basal diet + 100 mg magnolol additive/kg diet; MA200: basal diet + 200 mg magnolol additive/kg diet; MA300: basal diet + 300 mg magnolol additive/kg diet; CS30: basal diet + 30 mg antibiotic additive/kg diet.

### Serum and hepatic peroxide biomarkers

Comparing to the CS30 group, MA200 and MA300 significantly decreased the levels of 8-OHdG in serum (*P* = 0.043) (Table 7). And in the liver, MA200 and MA300 significantly decreased the levels of 8-OHdG (*P* = 0.007) and PC (*P* = 0.005) comparing to the control and CS30 groups. Albeit not significant, serum and hepatic MDA levels in three MA treated groups were lower than that in the control and CS30 groups (*P* = 0.721 and 0.392, respectively).

**Table 7:** Effect of different magnolol levels in diet on peroxide biomarkers of 28 d *Linwu* ducks.

Items^1^	C	MA100	MA200	MA300	CS30	SEM	*P*-value
Serum							
8-OHdG, ng/ml	10.48^ab^	10.19^ab^	9.93^b^	9.52^b^	11.23^a^	0.194	0.043
PC, nmol/mg prot	5.15	4.64	4.32	4.78	5.52	0.144	0.057
MDA, nmol/ml	3.55	3.34	3.41	3.45	3.61	0.063	0.721
Liver							
8-OHdG, ng/mg prot	32.81^ab^	29.68^bc^	25.99^c^	26.13^c^	35.88^a^	1.150	0.007
PC, nmol/mg prot	15.39^ab^	13.79^bc^	12.33^c^	11.69^c^	16.59^a^	0.541	0.005
MDA, nmol/mg prot	0.43	0.42	0.41	0.38	0.47	0.013	0.392

8-OHdG = 8-hydroxy-2’-deoxyguanosine; PC = protein carbonyl; MDA = malonaldehyde.

^a-c^In the same row, values with different small letter superscripts mean significant difference (*P* < 0.05).

^1^C: control, basal diet; MA100: basal diet + 100 mg magnolol additive/kg diet; MA200: basal diet + 200 mg magnolol additive/kg diet; MA300: basal diet + 300 mg magnolol additive/kg diet; CS30: basal diet + 30 mg antibiotic additive/kg diet.

### The mRNA expression levels of hepatic antioxidant-related genes

**Fig 2:**
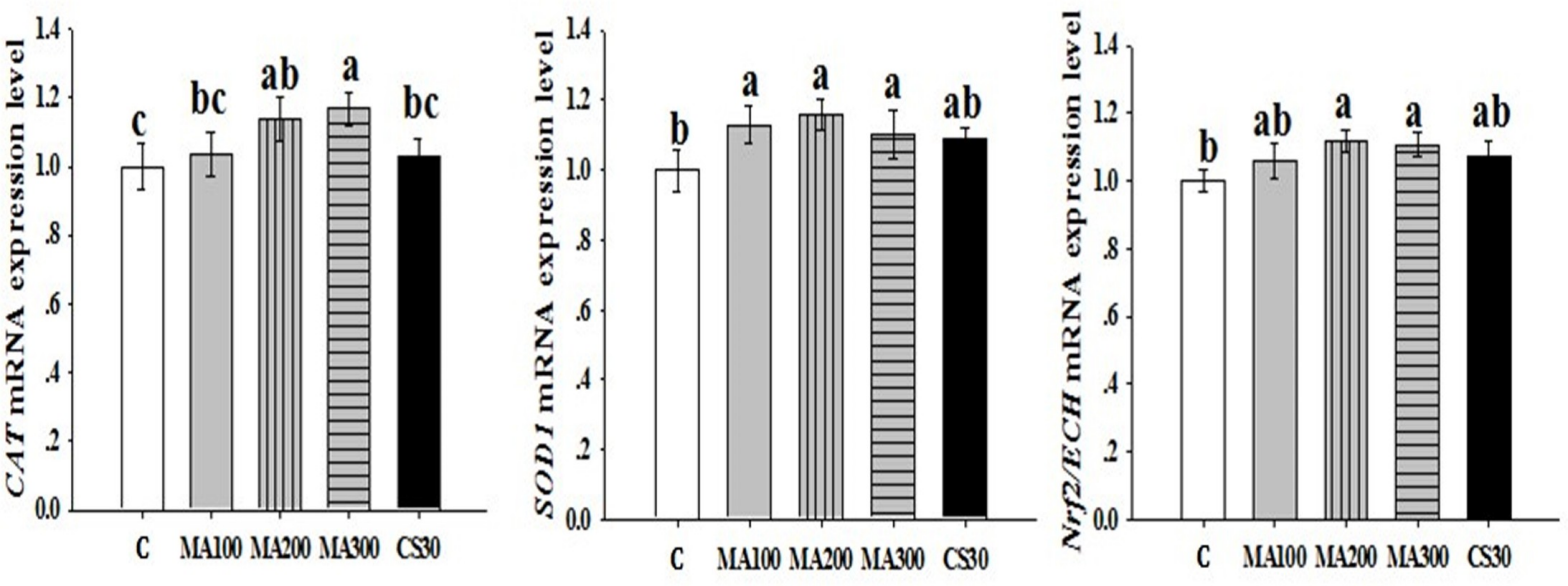
Effect of dietary magnolol at different levels on the mRNA expressions of hepatic antioxidant-related genes in 28 d *Linwu* ducklings. C: control, basal diet; MA100: basal diet + 100 mg magnolol additive/kg diet; MA200: basal diet + 200 mg magnolol additive/kg diet; MA300: basal diet + 300 mg magnolol additive/kg diet; CS30: basal diet + 30 mg antibiotic additive/kg diet.

Comparing to the control group, MA200 and MA300 significantly increased (*P* < 0.05) the *CAT*, *SOD1* and *Nrf2/ECH* mRNA expression levels (Fig 2). In addition, the *CAT* mRNA expression level was significantly higher (*P* < 0.05) in MA300 than that in CS30 (Fig 2). Moreover, there were no significant difference (*P* > 0.05) on expression levels of *MnSOD*, *GPX4*, *GSTα3*, *GPX1*, *HO-1* and *Keap-1* among each group (S1 Fig).

## Discussion

The early growing stage was important and challenging for *Linwu* ducks in the modern breeding system, as their capabilities of environment adaptation, nutrients digestion, and prevention to pathogenic infections were weak during the 7 to 28 d period of time [30]. Therefore, it was critical to prevent the stresses and diseases with dependable methods in ducklings at the early growing stage. The antibiotics were commonly used as feed additives because of the promotional function in livestock growth performance and preventive effect against pathogenic diseases. However, the abuse of antibiotic additives in animal feed caused many problems such as drug resistance. It was widely accepted that phytochemical had beneficial effects on livestock performance [31–33]. Average final weight, ADG, ADFI, and F/G ratio are important indices for growth performance evaluation. In the present experiment, it was found that intermediate and high level of MA improved the average final weight, ADG and F/G ratio of *Linwu* ducklings compared with the control group, which showed that MA could improve *Linwu* ducks’ growth performance. These findings were consistent with previous data [34]. Additionally, our data showed that the supplementation with CS also improved ducklings’ growth performance comparing to the control group. It was reported that the mechanisms of CS on poultry’s growth performance might be attributed to the bactericidal effect [35]. In this experiment, it was possible that magnolol modulated the architecture and the antioxidant capacity of the intestinal mucosal, which further enhanced the digestion, absorption and metabolism of nutriments, and finally increased the growth performance in *Linwu* ducklings.

The intact morphology of intestinal mucosa was one of the most important indications of the nutrient digestive and absorptive capacities in poultry [24]. The morphology condition could be represented by indicators such as villus height, crypt depth and the ratio between these two. Crypt depth was associated with the level of cell turnover, and the lengthening of the villus (villus height) increased the surface area for nutrient absorption [36]. Besides, the villi kept renewing to replace the sloughing onces, as well as the damaged ones caused by pathogenic assault and pathogen-initiated inflammation [37]. Therefore, the higher villus height/Crypt depth ratio indicated better digestive and absorptive capabilities. In the present study, it was found that MA significantly enhanced the villus height/Crypt depth ratio of ileum, which was similar to what was reported previously [34]. In addition, Mei [38] observed that magnolol pretreatment attenuated heat stress-induced injury in intestinal epithelial cell 6 (IEC-6) cells and maintained the intact structures and functions of small intestine, which further proved the protective and ameliorative function of magnolol on intestinal morphology. This positive effect of magnolol might be related to its antioxidant activity, which eliminated the reactive oxygen species and reactive nitrogen species that caused damage to the intestine morphology and function [39].

Serum glucose was mainly the dietary carbohydrates which were digested into blood to sustain the organism’s need for energy [40]. And serum total protein content reflected the organism’s protein metabolism. As the protein content increased, the absorption and utilization rate of amino acid were improved to some extent [41]. Our study showed significant increases in serum contents of glucose and total protein in the group receiving 200 or 300 mg/kg dietary magnolol, which implied that magnolol could improve the digestion and absorption of nutrients. These data are similar to previous finding that magnolol stimulate glucose uptake in skeletal muscle cell [42]. It was likely that the promotional effect of nutrition absorption was related to the protection function of magnolol on the intestinal mucosal morphologies. Moreover, previous studies showed that glucose was one of the indicators of stresses, especially heat stress, and its concentration decreased as the stress condition became severe [43, 44]. Therefore, it was possible that dietary magnolol relieved the stress causing the increase of serum content of glucose.

Oxidative stress was resulted from the overproduction of free radicals, for example superoxide anion (**O**_**2**_^**-**^) and nitric oxide radical (**NO•**). When the free radicals content exceeded the modulation capacity of antioxidant defense, it would cause problems such as lipid peroxidation, protein nitration, DNA damage and apoptosis. The oxidative stress broke the balance among the components of the gastrointestinal tract, and influenced the health status and productivity of poultry [39]. The antioxidant enzymes played critical roles in eliminating the notorious free radicals and maintaining the redox homeostasis. SOD was a major antioxidant enzyme which catalyzed the dismutation of the superoxide (O_2_^-^) radical into O_2_ and H_2_O_._ GPX was an enzyme family with activities of detoxifying peroxides and hydroperoxides [45]. Other than the function of scavenging the free radical which was contributed to the sulfhydryl, GSH was also a unique substrate for glutathione S-transferase and GPX, who were able to remove the free radicals and peroxides from cells [46]. Our study showed a significant increase in serum levels of SOD, GPX and GSH in MA200 and MA300, which meant magnolol could improve the antioxidative status of *Linwu* ducklings by increasing the levels of antioxidant enzymes. MDA, PC and 8-OHdG were also applied to evaluate the oxidative stress level on lipid, protein and nucleic acid respectively [47]. MDA was an end-product from a series of reactions during lipid peroxidation caused by ROS [47]. PC was the most common product of protein oxidation and derivation of arginine, proline, lysine and threonine [48]. 8-OHdG could be induced by DNA damage caused by oxidative stress, because ROS could react with dGTP in the nucleotide pool forming 8-OHdG during DNA replication [49]. Our data showed that magnolol decreased the levels of MDA, PC and 8-OHdG in serum or liver, which demonstrated that magnolol might modulate the antioxidant status of *Linwu* ducklings. Xia [50] found that magnolol suppressed oxidative stress in the small intestinal tissue of mice induced by oxaliplatin, which supported what was found in the present experiment. The mechanism of antioxidative effect with magnolol was uncertain. It was supposed that medium to high level of magnolol increased the expression of SOD, GPX, and GSH in serum and boosted the production of the antioxidative enzymes, which scavenged the free radicals and alleviated the oxidative stress.

To the contrary, other than the antioxidative effect of magnolol, it was found that ducklings fed with CS had increased levels of oxidative products such as 8-OHdG, PC or MDA in serum and liver. It suggested that long-term intake of CS caused large amounts of ROS production, resulting in an obvious oxidation of DNA, protein and lipid. Previous studies reported that CS had dose-dependent neurotoxicity and nephrotoxicity [51–53], which resulted in oxidative stress, apoptosis, and abnormal inducible nitric oxide synthase levels [54, 55]. Moreover, CS was reported to contribute to liver failure [51].

Nuclear factor erythroid-2-related factor 2 (**Nrf2**) was a key transcription factor that regulated the cell redox status by inducing the expression of detoxifying enzyme and antioxidant enzyme in cells [56]. CAT, GPX and SOD as the main antioxidant enzyme were downstream protein of Nrf2/antioxidant responsive element (**ARE**) pathway which were modulated by Nrf2 molecules [57]. Our study showed a significant increase in hepatic mRNA expression levels of *Nrf2/ECH* and *CAT*, *SOD1* in the group supplemented with 200 and 300 mg/kg magnolol, which meant magnolol might improve the antioxidative status of *Linwu* ducklings by activating Nrf2 and promoting ARE-mediated gene expressions. Previous study reported that magnolia bark extract activated Nrf2-dependent gene expression and protected against hydrogen peroxide mediated oxidative stress in hepatocytes. And it was identified that magnolol was the active phytochemical inducing the Nrf2-mediated activity [58]. Li et al. [59] also proved in zebrafish embryos that low dose of honokiol (0.6 and 0.9 ug/mL) increased the mRNA expression of Nrf2, SOD1, SOD2, catalase and heme oxygenase 1, resulting in decreasing content of ROS. It could be concluded that magnolol could induce the amelioration of oxidative stress in *Linwu* buck liver by activating the Nrf2/ARE pathway and triggering the expression of antioxidant protein SOD, GPX and GSH, thereby scavenging the overproduced ROS in the tissue and serum. These could be the molecular basis for the beneficial activities of magnolol in our study.

## Conclusion

In conclusion, dietary magnolol at 200 to 300 mg/kg showed promising effects on increasing the overall performance of *Linwu* ducklings by activating Nrf2/ARE pathway, increasing the levels of antioxidant enzymes, and improving intestinal morphology.

## Supporting information

S1 Fig Effect of different magnolol levels in diet on the hepatic expression levels of antioxidant-related genes of 28 d Linwu duck. C: control, basal diet; MA100: basal diet + 100 mg magnolol additive/kg diet; MA200: basal diet + 200 mg magnolol additive/kg diet; MA300: basal diet + 300 mg magnolol additive/kg diet; CS30: basal diet + 30 mg antibiotic additive/kg diet.
(DOCX)
