# Supplementary material for: Magnolol additive improves growth performance of Linwu ducklings by modulating antioxidative status
Source: PLoS One. 2021 Dec 31;16(12):e0259896. doi: 10.1371/journal.pone.0259896 (PMC8719751; doi:10.1371/journal.pone.0259896)
Supplement: S1 Fig — C: control, basal diet; MA100: basal diet + 100 mg magnolol additive/kg diet; MA200: basal diet + 200 mg magnolol additive/kg diet; MA300: basal diet + 300 mg magnolol additive/kg diet; CS30: basal diet + 30 mg antibiotic additive/kg diet. (DOCX) [file pone.0259896.s001.docx]

**S1 Fig. Effect of different magnolol levels in diet on the hepatic expression levels of antioxidant-related genes of 28 d Linwu duck.** C: control, basal diet; MA100: basal diet + 100 mg magnolol additive/kg diet; MA200: basal diet + 200 mg magnolol additive/kg diet; MA300: basal diet + 300 mg magnolol additive/kg diet; CS30: basal diet + 30 mg antibiotic additive/kg diet.
